# Antibiotic prescribing for care-home residents: a population-based, cross-classified multilevel analysis in Scotland, UK

**DOI:** 10.1093/ageing/afae288

**Published:** 2025-01-09

**Authors:** Nicosha De Souza, Bruce Guthrie, Suzanne Grant, Fabiana Lorencatto, Jane Dickson, Aleksandra Herbec, Carmel Hughes, Jacqueline Sneddon, Peter T Donnan, Charis A Marwick

**Affiliations:** School of Medicine, University of Dundee, Dundee, UK; Usher Institute Centre for Population Health Sciences, University of Edinburgh, Edinburgh, UK; School of Medicine, University of Dundee, Dundee, UK; Centre for Behaviour Change, University College London, London, UK; Institute-European Observatory of Health Inequalities, Calisia University, Kalisz, Wielkopolskie, Poland; School of Medicine, University of Dundee, Dundee, UK; Centre for Behaviour Change, University College London, London, UK; Institute-European Observatory of Health Inequalities, Calisia University, Kalisz, Wielkopolskie, Poland; School of Pharmacy, Queen’s University Belfast, Belfast, UK; British Society for Antimicrobial Chemotherapy, Birmingham, UK; School of Medicine, University of Dundee, Dundee, UK; School of Medicine, University of Dundee, Dundee, UK

**Keywords:** antibiotic, antimicrobial stewardship, antibiotic prescribing, care-homes, cross-classified, older people

## Abstract

**Background:**

There is wide variation in antibiotic prescribing across care-homes for older people, with implications for resident outcomes and antimicrobial resistance.

**Objective:**

To quantify variation in antibiotic prescribing and associations with resident, care-home and general practice characteristics.

**Design:**

Population-based analyses using administrative data.

**Setting and subjects:**

148 care-homes in two Scottish regions, with 6633 residents registered with 139 general practices.

**Methods:**

Prescriptions for any antibiotic and for broad-spectrum antibiotics between 1 April 2016 and 31 March 2017 were analysed using cross-classified multilevel negative binomial regression.

**Results:**

For any antibiotics, the mean prescription rate was 6.61 (SD 3.06) per 1000 resident bed-days (RBD). In multivariate analysis, prescribing was associated with resident age [incidence rate ratio (IRR) 1.30 [95% confidence interval 1.19 to 1.41] for 90+ versus <80 years old] and comorbidity (1.88 [1.71 to 2.06] for Charlson Comorbidity Index 3+ versus 0), and the care-home’s sampling rate for microbiological culture (1.53 [1.28 to 1.84] for >7 versus <3.5 samples per 1000 RBD), with residual unexplained variation between care-homes (median IRR 1.29 [1.23 to 1.36]) and general practices (1.11 [1.05 to 1.18]). For broad-spectrum antibiotics, the mean rate was 0.98 (0.92) per 1000 RBD. Broad-spectrum prescribing was also associated with resident age, sex, comorbidity and sampling rate, with larger residual unexplained variation between care-homes (1.56 [1.36 to 1.77]) and general practices (1.51 [1.31 to 1.72]).

**Conclusion:**

Variation in prescribing was influenced by resident case-mix, but there is significant unexplained variation between care-homes and between general practices, indicating a need for antibiotic stewardship to target both.

## Graphical Abstract

**Graphical Abstract:**
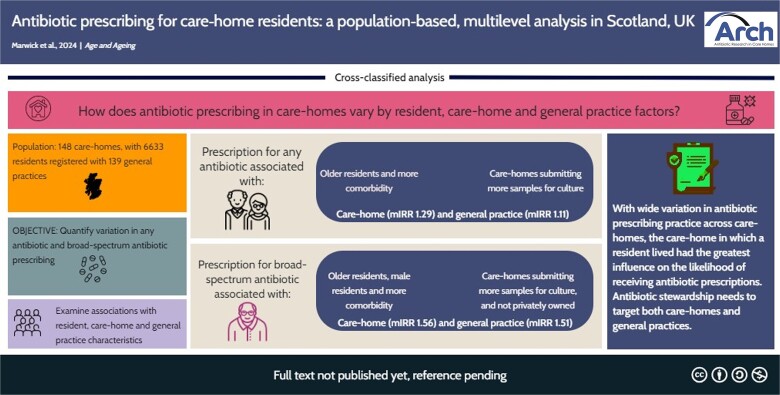

## Key Points

- In this population-based study, we found wide variation in antibiotic prescribing across care-homes for older people.
- There was significant residual unexplained variation in prescribing for residents by care-home and general practice, after accounting for specific resident, care-home and practice characteristics.
- Higher care-home rates of submission of resident samples (mostly urine samples) for microbiological culture was the main potentially modifiable factor associated with increased antibiotic prescribing.
- A general practice’s antibiotic prescribing rate overall was not associated with prescribing for care-home residents, indicating different influences in the care-home context.
- Antimicrobial stewardship interventions for care-home residents need to target behaviours within the care-home and general practice contexts.

## Introduction

Care-homes for older people have been brought into the spotlight by COVID-19 [1]. In the UK, care-homes support older people who can no longer live independently and include nursing homes, where staff include registered nurses, and residential homes. The vulnerability of care-home residents to infection and adverse outcomes, and a lack of resource prioritisation, are amongst issues that COVID-19 made painfully explicit [2], but which preceded the pandemic and have wider-reaching implications. Antibiotic resistance is a global public health threat with recent estimates of 1.27 million attributable deaths in 2019 [3]. Care-home residents have higher rates of antibiotic-resistant infections than older people living in their own homes [4–10]. Gram-negative coliform bacteria, which cause most urinary tract infections (UTIs) and bloodstream infections, are a particular concern [5, 8, 9].

In care-homes, antibiotic use varies within and between countries but up to 30% of residents receive antibiotics at any one time [11, 12] with concerns over prescribing quality [13]. Care-homes therefore need more antimicrobial stewardship, but evidence in this context is limited with systematic reviews including small numbers of studies [14–20]. Antimicrobial stewardship aims to (1) ensure effective prophylaxis and treatment of infection and (2) reduce unnecessary and potentially harmful antimicrobial use. Achieving both in care-homes is challenging because residents are vulnerable to infection [21], but also to adverse antibiotic effects including resistance and *Clostridioides difficile* infection [4–8, 10, 22, 23]. High antibiotic prescribing rates could be driven by characteristics of the care-home (such as excessive urine testing leading to unnecessary treatment of asymptomatic bacteriuria) [24] and/or of prescribers (such as being generally high-prescribers of antibiotics) [25, 26].

Published antibiotic prescribing rates often involve volunteer care-homes conducting cross-sectional point-prevalence surveys [11, 12]. Care-home residency is rarely reliably recorded in routine administrative datasets, precluding population-level and longitudinal analyses investigating the true extent of variation in antibiotic prescribing by care-home and by primary medical care provider. Studies accounting for clustering by care-home [27–29], prescriber [25, 26] or both [30] either report significant residual unexplained variation [25, 26, 28] or do not explicitly report variation [27, 30]. Such studies vary in inclusion of specific resident (e.g. age, comorbidity), care-home (e.g. size, staffing) and prescriber (e.g. prescribing history) characteristics. Only one study examined variation across all three while accounting for clustering, but residual variation by care-home or prescriber was not reported [30]. Potential sampling bias was a frequent limitation.

The aim of this study was to quantify prescribing rates of any antibiotics and broad-spectrum antibiotics for residents of all care-homes for older people in two National Health Service (NHS) health board regions in Scotland, and to quantify the relative contributions of resident, care-home and general practice (prescriber) characteristics in multilevel cross-classified models.

## Methods

### Population

All residents of care-homes for older people in two neighbouring NHS health board regions (estimated populations of 414 000 and 371 000, respectively) of Scotland, UK, between 1 April 2016 and 31 March 2017 were included. Data available for these regions (see ethics/approvals section) include some required datasets which are not available nationally. Residents were identified and allocated to specific care-homes initially using algorithms matching population addresses in the Scottish Master Community Health Index (CHI—individuals’ addresses from NHS general practice registration) with care-home addresses from the Scottish regulatory body, the Care Inspectorate [31, 32]. Addresses flagged by algorithms underwent dual-operator manual checking.

### Data and sources

Resident demography (including general practice registration) was linked to community dispensed prescribing, hospital admissions, deaths and microbiology using CHI (used across all NHS services). Publicly available data were extracted from Care Inspectorate registrations and reports [32], care-home websites, general practice reports from Public Health Scotland [33] and the 2017/18 NHS Scotland Health and Care Experience Survey [34]. See Appendix 1 in Supplementary Data for visual representation of data sources and linkage.

### Outcomes

Outcomes were (1) antibiotic prescriptions, defined as prescribed items dispensed in the community for systemic drugs included in the British National Formulary [35] chapter 5.1 (Antibacterials) and available orally, excluding those listed in Appendix 2 (Supplementary Data); and (2) broad-spectrum antibiotic prescriptions which have activity against Gram-negative coliform bacteria and are not first-line guideline-recommended therapy in the community in study regions due to higher associations with antibiotic resistance, and other adverse effects, than recommended first-line antibiotics. These included cephalosporins, co-amoxiclav, fluoroquinolones, fosfomycin and pivmecillinam. Prescription rates were calculated per 1000 resident bed-days (RBD).

### Resident characteristics

Resident characteristics included age (<80, 80–89, 90+ years old), sex, diagnosis of dementia (see Appendix 2 in Supplementary Data) and other comorbidity [Charlson Comorbidity Index (CCI) [36] from the International Classification of Diseases, Tenth Revision [37] hospital discharge codes], with dementia excluded.

#### Care-home characteristics

Care-home characteristics included residential versus nursing (nursing care provided by care-home staff), number of beds (<25, 25–40, >40), rurality of location (urban, small town, rural) [38], ownership (private versus local authority or not-for-profit/voluntary), number of general practices with residents registered and culture sample submission rate (samples submitted during routine care to an NHS Microbiology Laboratory for culture, per 1000 RBD). Care Inspectorate reports [32] provided grading on quality of (1) staffing, (2) management and leadership, (3) care and support and (4) environment (each six-point scales, categorised as 1–3 = weak, 4 = good, 5–6 = excellent), an overall quality rating (mean of the four individual scores) and any complaints about the care-home upheld and/or ‘enforced requirements’ (changes mandated following inspection) in the last 3 years.

#### General practice characteristics

General practice characteristics included number of registered patients, proportion of all registered patients aged ≥65 years, deprivation (percentage of all registered patients living in the 15% most deprived postcodes in Scotland), total antibiotic prescribing rate (per 1000 registered patients per year) and patient satisfaction, defined as the proportion of national patient experience survey respondents rating practice’s overall care as excellent or good (<80%, 80 to 89%, 90 + %) [34].

### Statistical analysis

The data have a cross-classified structure, where residents of one care-home can be registered with multiple general practices, and general practices can care for residents of multiple care-homes. See Appendix 3 in Supplementary Data for visual representation of data structure. Cross-classified multilevel negative binomial regression models were used to estimate incidence rate ratios (IRRs) for the likelihood of a resident having any antibiotic prescription, and a broad-spectrum antibiotic prescription, associated with resident, care-home and general practice characteristics (fixed effects), and examine variation in prescribing to residents between care-homes and between general practices using the median IRR (mIRR) which expresses variation (random effects) between higher-level units on the same scale as fixed effects [39]. Each resident, care-home and general practice characteristic was examined in a univariate multilevel model. Multivariate analyses included variables significant (*P* < .05) in univariate analyses and any significant two-way interactions between variables. Data management and analyses used Stata version 17 (StataCorp LLC).

### Ethics and approvals

The University of Dundee Health Informatics Centre (HIC) Trusted Research Environment (TRE) [40] has ISO27001 accreditation and approval from the East of Scotland NHS Research Ethics Committee. Studies conducted by approved researchers that follow HIC Standard Operating Procedures and only analyse anonymised data in the HIC TRE do not need further individual study review. Manual address validation was approved by the NHS Caldicott Guardian (Reference: GTCAL4179).

## Results

In total, 6633 residents of 148 care-homes contributed 4558 person-years of observation. Their mean (SD) age was 84.7 (8.9) years, 69% were female and 47% had dementia (Table 1). Half of the care-homes provided nursing care and the mean number of beds was 38.1 (17.0). Almost two-thirds were urban, with 61% in the larger health board region, the majority (80%) privately owned and most having high quality ratings. There were 6777 (4855 [72%] urine) samples submitted for culture with a mean 5.63 (3.4) per 1000 RBD across care-homes. Nearly a third of care-homes had residents registered with more than eight general practices. Of 139 general practices, around half had at least 20% registered patients aged 65+ years, and most issued >600 antibiotic prescriptions per 1000 total registered patients per year (Table 1)*.*

**Table 1:** Distribution of resident, care-home and general practice characteristics, and univariate associations with the likelihood of residents receiving a prescription for any antibiotic, and for a broad-spectrum antibiotic, from negative binomial cross-classified models

Variable	Category (*N*)	Association with any antibiotic prescription		Association with broad-spectrum antibiotic prescription	
		IRR (95% CI)	*P*-value	IRR (95% CI)	*P*-value
**Residents**	***N* = 6633**				
Age (years)	<80 (1498)	Baseline	<.001	Baseline	.02
	80 to 89 (2973)	1.12 (1.03–1.21)		1.23 (0.99–1.54)	
	90+ (2162)	1.28 (1.17–1.40)		1.41 (1.12–1.79)	
Sex	Female (4577)	Baseline	.11	Baseline	.01
	Male (2056)	1.06 (0.99–1.13)		1.28 (1.07–1.53)	
Dementia	No (3539)	Baseline	.37	Baseline	.36
	Yes (3094)	1.03 (0.97–1.10)		1.08 (0.91–1.28)	
Charlson Comorbidity Index excluding dementia	0 (4216)	Baseline	<.001	Baseline	<.001
	1 to 2 (1571)	1.45 (1.34–1.56)		1.78 (1.47–2.16)	
	3+ (846)	1.86 (1.70–2.05)		2.57 (2.02–3.26)	
**Care-homes**	***N* = 148**				
Care type	Nursing (72)	Baseline	.01	Baseline	.22
	Residential (76)	1.17 (1.03–1.32)		1.17 (0.91–1.51)	
Size (number of beds)	<25 (41)	Baseline	.18	Baseline	.17
	25 to 40 (58)	0.97 (0.82–1.14)		1.12 (0.79–1.59)	
	>40 (49)	0.87 (0.74–1.03)		0.85 (0.60–1.20)	
Rurality	Urban (94)	Baseline	.27	Baseline	.08
	Small town (28)	0.96 (0.80–1.14)		0.73 (0.50–1.08)	
	Rural (26)	0.86 (0.72–1.03)		0.68 (0.46–1.02)	
Health board	A (90)	Baseline	.01	Baseline	.18
	B (58)	1.20 (1.05–1.38)		1.23 (0.91–1.68)	
Ownership	Private (118)	Baseline	<.001	Baseline	<.001
	Other^a^ (30)	1.35 (1.15–1.57)		1.84 (1.36–2.48)	
General practices with residents registered	1 to 3 (46)	Baseline	.86	Baseline	.89
	4 to 8 (60)	0.99 (0.84–1.16)		0.94 (0.67–1.33)	
	>8 (42)	0.95 (0.80–1.13)		1.01 (0.70–1.47)	
Quality overall^b^ (mean across measures)	Weak (21)	Baseline	.99	Baseline	.47
	Good (50)	1.01 (0.83–1.24)		1.21 (0.80–1.82)	
	Excellent (77)	1.01 (0.84–1.21)		1.27 (0.86–1.88)	
Quality: management and leadership^c^	Weak (28)	Baseline	.74	Baseline	.39
	Good (49)	1.05 (0.88–1.26)		1.29 (0.90–1.86)	
	Excellent (71)	0.99 (0.84–1.18)		1.18 (0.83–1.67)	
Quality: staffing^c^	Weak (25)	Baseline	.76	Baseline	.70
	Good (49)	0.94 (0.77–1.14)		0.94 (0.64–1.39)	
	Excellent (74)	0.94 (0.78–1.12)		1.07 (0.75–1.53)	
Quality: environment^c^	Weak (20)	Baseline	.45	Baseline	.53
	Good (57)	0.92 (0.75–1.12)		1.05 (0.71–1.55)	
	Excellent (71)	1.00 (0.82–1.21)		1.19 (0.82–1.75)	
Quality: care and support^c^	Weak (26)	Baseline	.45	Baseline	.50
	Good (55)	0.94 (0.79–1.13)		1.10 (0.76–1.59)	
	Excellent (67)	1.03 (0.87–1.23)		1.23 (0.86–1.75)	
Complaint(s) upheld in last 3 years	No (66)	Baseline	.01	Baseline	.56
	Yes (82)	0.85 (0.75–0.97)		0.93 (0.72–1.20)	
Enforced requirement^d^ in last 3 years	No (90)	Baseline	.82	Baseline	.40
	Yes (58)	0.98 (0.86–1.12)		0.89 (0.68–1.16)	
Culture sample submission rate (per 1000 RBD)^e^	<3.5 (50)	Baseline	<.001	Baseline	<.001
	3.5 to 7 (50)	1.12 (0.98–1.29)		1.22 (0.90–1.65)	
	>7 (48)	1.56 (1.36–1.80)		2.06 (1.49–2.84)	
**General practices**	***N* = 139**				
Number of registered patients^6^	<5000 (39)	Baseline	.58	Baseline	.94
	5–10 000 (65)	1.02 (0.89–1.15)		1.01 (0.70–1.44)	
	>10 000 (11)	1.09 (0.91–1.30)		0.93 (0.56–1.56)	
Percentage of registered patients aged 65+ years^7^	<20 (59)	Baseline	.86	Baseline	.82
	20+ (56)	0.99 (0.89–1.10)		0.97 (0.72–1.30)	
Deprivation (% of patients in 15% most deprived postcodes)^8^	0 to 15 (73)	Baseline	.24	Baseline	.11
	>15 (42)	1.08 (0.95–1.23)		1.30 (0.94–1.78)	
Antibiotic prescriptions (per 1000 patients per year)^9^	≤600 (51)	Baseline	.28	Baseline	.26
	>600 (70)	1.06 (0.95–1.17)		1.19 (0.88–1.60)	
Patient satisfaction (% positive about overall care)^10,11^	<80 (36)	Baseline	.27	Baseline	.82
	80 to 89 (41)	0.95 (0.84–1.08)		0.99 (0.70–1.41)	
	90+ (37)	0.90 (0.79–1.02)		0.90 (0.61–1.31)	

^a^Other ownership = local authority or voluntary service.

^b^Overall quality = mean of four quality measures (each out of 6): weak <3.5; good 3.5 to <4.5; excellent ≥4.5.

^c^Individual quality measures (out of 6): weak = 1, 2, 3; good = 4; excellent = 5, 6.

^d^Changes mandated following inspection by the Care Inspectorate.

^e^RBD, resident bed-days.

^f^GP practice data were missing for 24^6,7,8^, 18^9^ and 25^10^ practices.

^g^Percentage (weighted) of respondents rating the overall care provided by their GP practice as Excellent or Good.

A total of 14 977 antibiotic prescriptions were dispensed to 4306 (65%) residents, with a mean 1.62 (SD 2.49 range 0–25) per resident, in the study year. The most frequently prescribed was amoxicillin (25% of all prescriptions). Also, 1562 broad-spectrum antibiotics were dispensed to 878 (13%) residents, with a mean 0.24 (SD 0.90, 0–16) per resident. See Appendix 4 in Supplementary Data for frequencies of antibiotics and residents receiving them in the study year.

**Figure 1:**
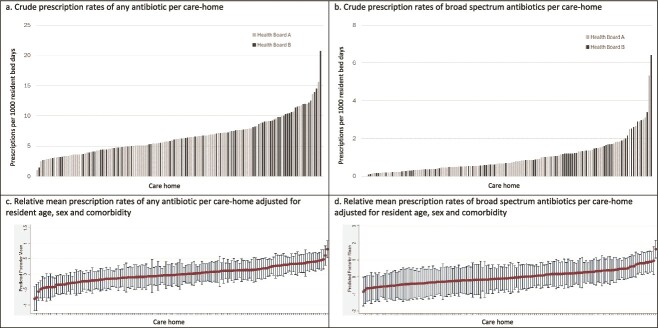
Mean antibiotic prescribing rates per 1000 resident bed-days per care-home over the study year as (a) crude rate of any antibiotic prescription; (b) crude rate of broad-spectrum antibiotic prescriptions; (c) relative mean rate of any antibiotic prescription adjusted for resident age, gender and comorbidity; and (d) relative mean rate of broad-spectrum antibiotic prescriptions adjusted for resident age, gender and comorbidity.

There was significant variation in prescribing by care-home with mean 6.61 (SD 3.06, range 1.04 to 20.72) prescriptions per 1000 RBD for any antibiotics (Figure 1a) and 0.98 (SD 0.92, 0 to 6.41) for broad-spectrum antibiotics (Figure 1b). Variation between care-homes persisted after adjustment for resident age, sex and CCI (Figure 1c and1d).

For any antibiotic prescription, empty regression models (with no resident, care-home or general practice characteristics) had mIRR between care-homes of 1.40 [95% confidence interval (CI) 1.33 to 1.49] and between general practices of 1.12 (1.03 to 1.19). In univariate models with care-home and general practice as cross-classified random variables, increased resident age (IRR 1.28 [1.17 to 1.40] for 90+ vs <80 years old) and comorbidity (1.86 [1.70 to 2.05] for CCI 3+ vs 0) were associated with increased likelihood of prescription. Other associations with care-home characteristics included residential care (1.17 [1.03 to 1.32] vs nursing), health board B (1.20 [1.05 to 1.38] vs A), ownership, complaint(s) upheld and culture sample submission rates (1.56 [1.36 to 1.80] for >7 vs <3.5 per 1000 RBD). No general practice characteristics were associated with prescribing, including practices’ overall antibiotic prescribing rates (Table 1).

In the adjusted model, resident age and comorbidity, and care-home culture sample submission rate (IRR 1.53 [95% CI 1.28 to 1.84] for >7 vs <3.5 per 1000 RBD) remained significant (Table 2). There was significant residual unexplained variation in antibiotic prescribing between care-homes (mIRR 1.29 [1.23 to 1.36]), similar in size to the IRR for resident age 90+ years versus <80 years. There was also significant variation between general practices, but at a lower level (mIRR 1.11 [1.05 to 1.18]), similar in size to the IRR for resident age 80–89 versus <80 years (Table 2).

**Table 2:** Adjusted associations between resident and care-home characteristics (with significant univariate associations) and the likelihood of residents receiving a prescription for any antibiotic, from multivariate negative binomial cross-classified models

Variable	Category	Association with ANY antibiotic prescription		Care-home median IRR (95% CI)	GP practice median IRR (95% CI)
		IRR (95% CI)	*P*-value		
Age (years)	<80	Baseline	<.001	1.29 (1.23–1.36)	1.11 (1.05–1.18)
	80 to 89	1.10 (1.01–1.19)			
	90+	1.30 (1.19–1.41)			
Charlson Comorbidity Index excluding dementia	0	Baseline	<.001		
	1 to 2	1.45 (1.34–1.56)			
	3+	1.88 (1.71–2.06)			
Care-home type	Nursing	Baseline	.89		
	Residential	0.99 (0.88–1.12)			
Health board	A	Baseline	.33		
	B	0.93 (0.80–1.08)			
Care-home ownership	Private	Baseline	.22		
	Other^a^	1.10 (0.94–1.28)			
Care-home complaint(s) upheld in last 3 years	No	Baseline	.13		
	Yes	0.92 (0.82–1.03)			
Culture sample submission rate (per 1000 RBD)^b^	<3.5	Baseline	<.001		
	3.5 to 7	1.12 (0.97–1.29)			
	>7	1.53 (1.28–1.84)			

^a^Other ownership = local authority or voluntary service.

^b^RBD = resident bed-days.

For broad-spectrum antibiotics, empty models had mIRR between care-homes of 1.73 (95% CI 1.53 to 1.99) and between general practices of 1.52 (1.31 to 1.78). In univariate models, increased resident age (IRR 1.41 [1.12 to 1.79] for 90+ vs <80 years old), male sex (1.28 [1.07 to 1.53]) and comorbidity (2.57 [2.02 to 3.26]) for CCI 3+ versus 0 were associated with increased likelihood of prescription. Associated care-home characteristics included ownership and culture sample submission rate (2.06 [1.49 to 2.84] for >7 vs <3.5 per 1000 RBD). No general practice characteristics were associated with broad-spectrum prescription (Table 1). In the adjusted model, the same resident and care-home characteristics remained significant (Table 3). Residual unexplained variation was similar between care-homes (mIRR 1.56 [1.36 to 1.77]) and between general practices (1.51 [1.31 to 1.72]) and larger than for any antibiotic prescription (Table 3).

**Table 3:** Adjusted associations between resident and care-home characteristics (with significant univariate associations) and the likelihood of residents receiving a prescription for a broad-spectrum antibiotic, from multivariate negative binomial cross-classified models

Variable	Category	Association with broad-spectrum antibiotic prescription		Care-home median IRR (95% CI)	GP practice median IRR (95% CI)
		IRR (95% CI)	*P*-value		
Age (years)	<80	Baseline	.01	1.56 (1.36–1.77)	1.51 (1.31–1.72)
	80 to 89	1.27 (1.02–1.59)			
	90+	1.48 (1.16–1.89)			
Sex	Female	Baseline	.03		
	Male	1.22 (1.02–1.46)			
Charlson Comorbidity Index excluding dementia	0	Baseline	<.001		
	1 to 2	1.72 (1.42–2.09)			
	3+	2.46 (1.93–3.12)			
Care-home ownership	Private	Baseline	.01		
	Other^a^	1.48 (1.11–1.98)			
Culture sample submission rate (per 1000 RBD)^b^	<3.5	Baseline	.004		
	3.5 to 7	1.15 (0.86–1.54)			
	>7	1.68 (1.22–2.32)			

^a^Other ownership = local authority or voluntary service.

^b^RBD = resident bed-days.

## Discussion

In this population-based cohort, we found significant variation in antibiotic prescribing across care-homes, both for any prescription and broad-spectrum prescriptions. Resident characteristics explained some of the variation, but there was significant residual unexplained variation between care-homes and between general practices which was larger for broad-spectrum prescriptions. Apart from culture sample submission rates, the specific individual care-home characteristics examined had little influence on antibiotic prescribing. However, the care-home in which a resident lived (as a higher-level variable) had more influence overall on whether residents received any antibiotic prescription than the general practice with which they were registered, and care-home and general practice had almost equal influence on whether residents received broad-spectrum antibiotic prescriptions. There was no association between antibiotic prescribing for care-home residents and general practices’ total antibiotic prescribing rates.

A key strength of this study is the inclusion of all residents of all care-homes for older people in a defined geographical area. Linkage across datasets using the CHI unique identifier enabled examination of relationships between care-homes and general practices, and applying cross-classified multilevel models enabled quantification of their relative contributions to antibiotic prescribing. We quantified residual variation (between care-homes and general practices) which was a similar size to some associations with resident characteristics. The overall population of the study region is representative of the Scottish population [33], and study care-homes are broadly similar to those across Scotland although slightly smaller (mean 38.1 vs 41.6 beds) with more privately owned (80% vs 71%). See Appendix 5 in Supplementary Data for comparison. A national programme of antibiotic stewardship includes all Scottish NHS health boards [41]. Data limitations include that general practice consultation data were not available, preventing assessment of appropriateness of prescriptions. Hospital antibiotic prescription data were also not available so any influence of receiving antibiotics in hospital on community prescriptions would be missed.

A further potential limitation is that the data analysed are from before the COVID-19 pandemic missing any potential impact on antibiotic prescribing. The time period analysed was just prior to a programme of work informed by higher-level analysis of the data. It is not straightforward to reproduce the presented analyses using more recent data due to the necessary preparation and curation of the data. This includes, but is not limited to, manual validation of individual-level address data and mapping of previous to current registration for care-homes which have changed management. These steps are required because care-home resident status, particularly *which* care-home, is still not routinely recorded in administrative datasets. We could not find more recent relevant published data, consistent with the complexity of data acquisition, curation and analysis. High-level data from 2020 including 129 of the 148 study care-homes demonstrate remarkably similar variation, with a mean of 6.78 (SD 3.03) antibiotic prescriptions per 1000 RBD, compared to 6.61 (3.06) in the study year. See Supplementary Data in Appendix 6 for additional comparison between these time periods. It seems highly unlikely that antibiotic prescribing in, and variation between, care-homes has reduced to a level where there is no need for further stewardship activity, or that the factors associated with variation have changed dramatically. Care-homes have been highlighted in the 2024 UK Antimicrobial Resistance National Action Plan as an important focus that has often been overlooked [42]. Antibiotic prescribing more generally in primary care in Scotland has fluctuated over time without a consistent decline in rates [43] despite ongoing stewardship activities [41].

Our observed antibiotic prescribing rates at resident level were lower than reported elsewhere, with 65% of residents receiving a prescription during the study year compared to 78% over 1 year [26] and 42% over 6 months [27], and a mean of 1.62 prescriptions per resident compared to 2.68 [28]. At care-home level, our mean 6.61 prescriptions per 1000 RBD was within the range reported across care-homes in one study of 73 US care-homes [27] and reasonably consistent with another study of 135 UK care-homes [28]. Point-prevalence surveys in Europe report that 4.9% to 30.6% of residents are receiving antibiotics at any one time [11, 12, 29], demonstrating wide variation similar to this study. Our findings are likely broadly generalisable to other contexts which may have even greater variation.

A relatively recent UK study accounting for clustering by care-home reported considerable variation in prescribing [28] but little association with specific care-home characteristics (microbiology sample submission rates were not included). An older US study accounted for clustering but did not report residual variation or care-home-level characteristics [27]. An Irish study reported significant clustering by care-home and that most characteristics reflecting active antibiotic stewardship were associated with lower antibiotic use except that residents of care-homes where samples were reportedly taken before starting antimicrobials had more antibiotic prescribing [29], consistent with our observed association between sampling and prescribing. Resident characteristics associated with antibiotic prescriptions in these studies included increased comorbidity [27, 28] and age [28], similar to our study, and the use of urinary catheters [27, 29]. There were no associations between sex and any antibiotic prescription, reflecting our finding. Our observed association between male sex and broad-spectrum antibiotics (not examined by others) may reflect UTI in men more often being complicated, with broader-spectrum antibiotics indicated in prescribing guidelines [44, 45].

Examining prescribing for care-home residents by prescriber (but not by care-home), two Canadian studies reported significant clustering of prescription start, duration and choice [25, 26]. Prescribers’ antibiotic prescribing practice overall was the dominant factor, more influential than resident characteristics [25]. This is consistent with our observed clustering by general practice, although we found no association with general practice overall antibiotic prescribing. One Northern Irish population-based study of associations between place of residence and antibiotic prescriptions amongst older adults accounted for clustering by both care-home and prescriber. They identified resident characteristics associated with higher prescribing (age, female sex and urinary catheters), but did not report residual variation between care-homes or general practices [30]. The association with female sex, contrasting our findings, was for the whole older population, adjusted for residence in a care-home (vs own home), and broad-spectrum antibiotics were not examined.

Our observed association between higher care-home culture sample (mostly urine) submission rates and residents receiving antibiotic prescriptions indicates a potentially modifiable stewardship intervention target [24]. High rates could indicate appropriate sampling and treatment, but this would raise concerns around infection prevention and control in affected care-homes. More likely, they indicate inappropriate sampling for nonspecific symptoms and asymptomatic bacteriuria (or other bacterial colonisation) with positive culture results prompting unnecessary antibiotic prescriptions [24]. Both scenarios indicate that infection prevention, diagnosis and management, including UTI, are key areas for stewardship in care-homes [44–46].

Our findings indicate that stewardship interventions need to target both care-home staff and prescribers. Nearly a third of care-homes having residents registered with more than eight general practices was striking and against recent policy recommendations [47], but was not associated with antibiotic prescription rates. Also notable was the lack of association between practice antibiotic prescription rates to all patients and to care-home residents, which is consistent with care-home prescribing having specific influences. Care-home antibiotic prescribing is usually done by a general practitioner, but increasingly by nurse practitioners [48] and pharmacists [49], and it involves behaviours beyond writing the prescription. Issuing a prescription is influenced by (1) decision to contact prescriber and/or submit a sample (care-home), (2) decision to prescribe (prescriber) and (3) choice of antibiotic [prescriber, influenced by culture result (care-home)]. Defining which and whose behaviours are involved at each stage, and the influential factors, is necessary to explore and explain variation and identify specific targets for tailored stewardship interventions. Quantitative studies rarely include behaviours or practices which are potentially amenable to intervention, with exceptions including submitting samples [29], the use of urinary catheters [27, 29, 30] and prescribers’ historical practice [25, 26].

A meta-synthesis of qualitative research including eight studies [50] and two additional studies [51, 52] examined factors influencing care-home antibiotic prescribing. Healthcare professionals and administrators identified variation in knowledge and practice, the unique care-home context (complex patients, limited access to doctors and diagnostic tests) and social factors (interactions between nurses, residents’ families and doctors) as influencing prescribing decisions [50–52]. While providing useful insights, the perspectives of residents/families and non-clinically trained care-home staff were not included. There is a need to expand on the limited qualitative evidence including their perspectives. This should incorporate ethnographic and behavioural science approaches, to give a fuller understanding of sociocultural influences, including on unexpected findings, e.g. our observed lower broad-spectrum antibiotic prescribing in privately owned care-homes. Such qualitative case studies form part of the broader Antibiotic Research in Care-Homes (ARCH) programme [53].

In this population-based study demonstrating wide variation in antibiotic prescribing practice across care-homes, the care-home in which a resident lived had the greatest influence on the likelihood of receiving antibiotic prescriptions, with the rate of sampling for culture an important potentially modifiable factor. The findings reinforce the need for further targeted antibiotic stewardship in both care-homes and general practices, and more research and innovation, engaging with care-home staff and prescribers.

## Supplementary Material

aa-24-1833-File003_afae288

## Research Data Transparency and Availability:

Data from this study are not publicly available but may be accessed by approved researchers via application to the Health Informatics Centre at the University of Dundee.
