# Supplementary material for: Antibiotic prescribing for care-home residents: a population-based, cross-classified multilevel analysis in Scotland, UK
Source: Age Ageing. 2025 Jan 9;54(1):afae288. doi: 10.1093/ageing/afae288 (PMC11711478; doi:10.1093/ageing/afae288)
Supplement: aa-24-1833-File003_afae288 [file aa-24-1833-file003_afae288.pdf]

Antibiotic prescribing for care-home residents: a population-based, cross-classified multilevel analysis in Scotland, UK: Supplementary Data

<u>Contents:</u>

Appendix 1. Data sources and linkage

Appendix 2. Definitions for Methods

Appendix 3. Structure of the multilevel cross-classified regression models

Appendix 4. Number of prescriptions and number of care-home residents with at least one prescription for community dispensed antibiotic prescriptions in the study year

Appendix 5: Number of beds and ownership of care-homes for older people in study health boards, and whole of Scotland for comparison (October 2017)

Appendix 6: Crude mean total antibiotic prescribing rates per 1000 resident bed days per care-home (a) over the study year April 2016 to March 2017, and (b) over 2020 (calendar year)

Appendix 1. Data sources and linkage

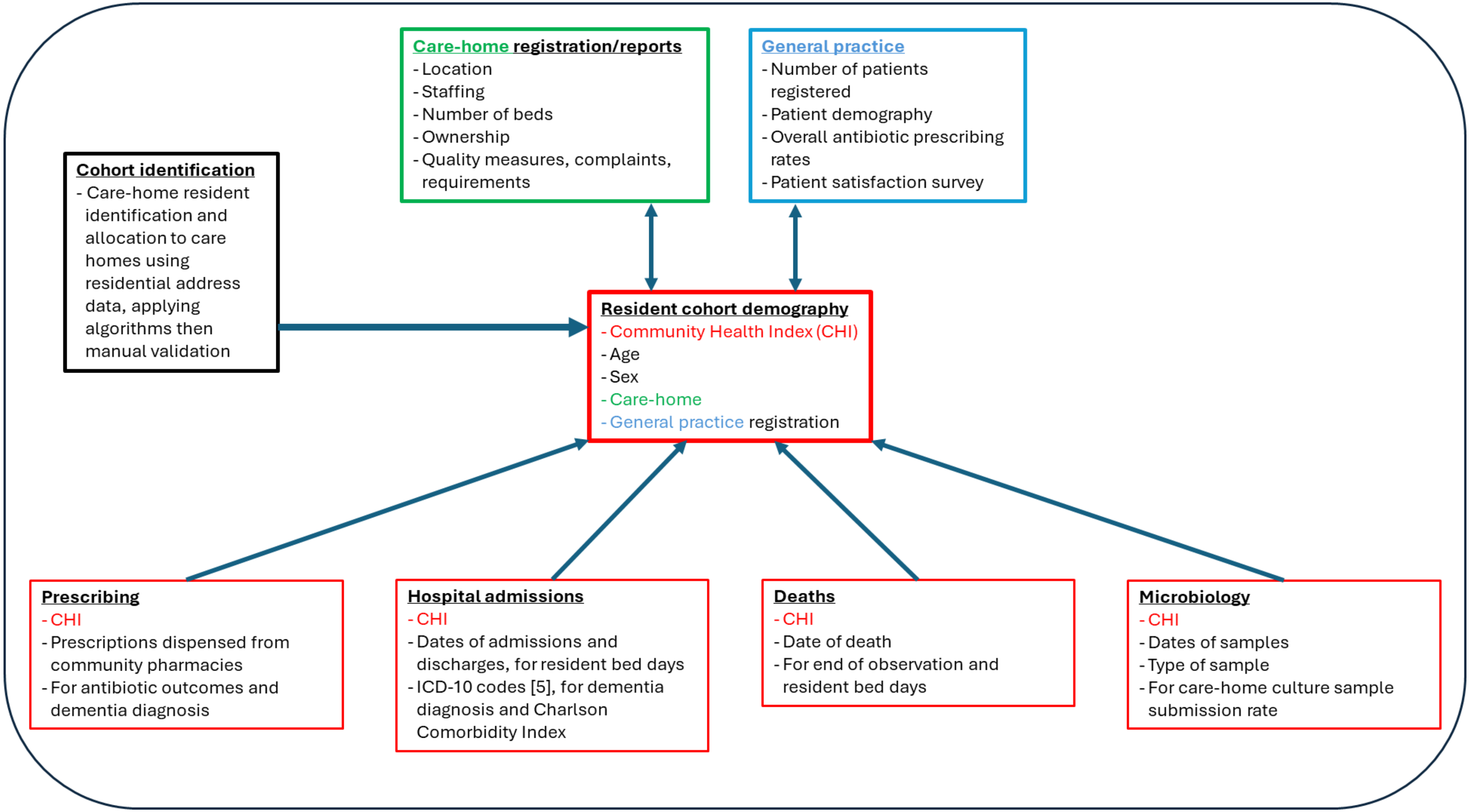

Appendix 2. Definitions for Methods

1. Excluded antibiotics: antibiotics for tuberculosis, leprosy, and those which, in the UK, are almost exclusively used for long-term skin conditions (lymecycline, minocycline, oxytetracycline) or non-infective conditions (demeclocycline hydrochloride, rifaximin).
2. Diagnosis of dementia: prescription of a dementia drug [memantine, galantamine, donepezil or rivastigmine] and/or a dementia hospital discharge code [International Classification of Diseases, Tenth Revision (ICD-10) [1] codes F00, F01, F02, F03, F051, G30 and G311].

Appendix 3. Structure of the multilevel cross-classified regression models

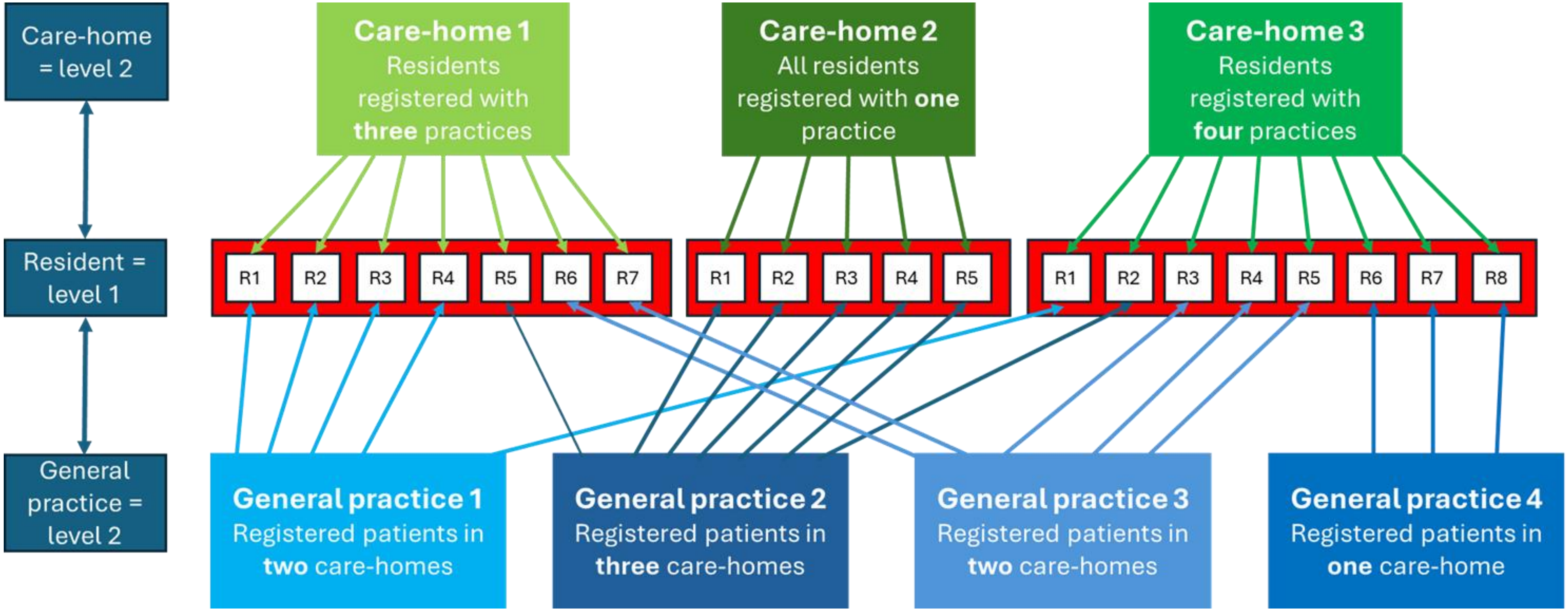

Appendix 4. Number of prescriptions and number of care-home residents with at least one prescription for community dispensed antibiotic prescriptions in the study year. Broad-spectrum antibiotics are in bold text.

ANTIBIOTIC APPROVED NAME	NUMBER OF PRESCRIPTIONS IN STUDY YEAR	NUMBER OF RESIDENTS WITH PRESCRIPTION IN STUDY YEAR
Amoxicillin	3725	2262
Trimethoprim	3060	1706
Nitrofurantoin	2286	1098
Doxycycline	1505	928
Flucloxacillin	1486	887
Clarithromycin	638	411
**Co-amoxiclav**	**577**	**424**
**Pivmecillinam**	**428**	**239**
**Ciprofloxacin**	**335**	**241**
**Cefalexin**	**238**	**97**
Metronidazole	204	163
Phenoxymethylpenicillin	124	93
Co-trimoxazole	118	71
Azithromycin	108	27
Erythromycin	77	52
Fusidic acid	14	<5*
Clindamycin	13	6
**Fosfomycin**	**12**	**11**
**Ofloxacin**	**7**	**6**
**Levofloxacin**	**6**	**<5***
**Cefuroxime**	**5**	**5**
Vancomycin	5	5
**Cefaclor**	**<5***	**<5***
Fidaxomicin	<5*	<5*
Linezolid	<5*	<5*
**Moxifloxacin**	**<5***	**<5***

* Numbers below five are suppressed to comply with data governance regulations

Appendix 5: Number of beds and ownership of care-homes for older people in study health boards, and whole of Scotland for comparison (October 2017 data from Care Inspectorate [2])

Region		Study health board regions	All Scotland
Total number of care-homes		148	878
Mean number of beds (SD)		38.1 (17.0)	41.6 (24.0)
Number of beds (%)	<25	41 (28%)	222 (25%)
	25 to 40	58 (39%)	258 (29%)
	>40	49 (27%)	360 (41%)
	Missing data	-	38 (4%)
Ownership (%)	Private	118 (80%)	621 (71%)
	Local authority or voluntary	30 (20%)	257 (29%)

Appendix 6: Crude mean total antibiotic prescribing rates per 1000 resident bed days per care-home (a) over the study year April 2016 to March 2017, and (b) over 2020 (calendar year)

<table>
<tr><td>a. Crude prescription rates of any antibiotic per care-home in 2016/17 study year
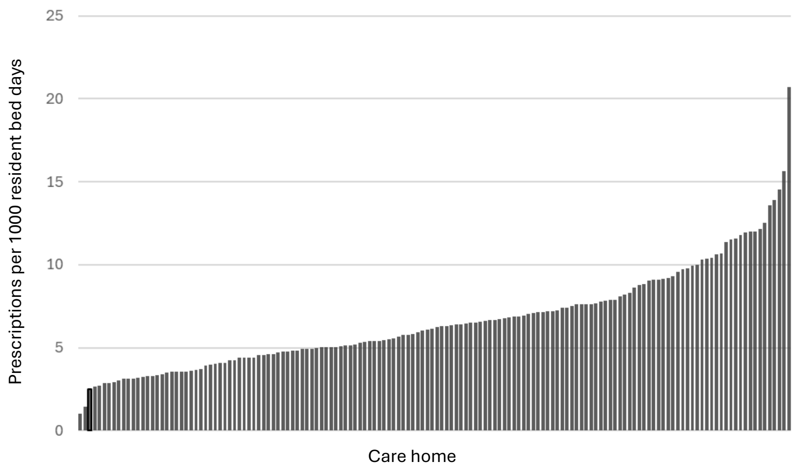
</td><td>b. Crude prescription rates of any antibiotic per care-home in 2020
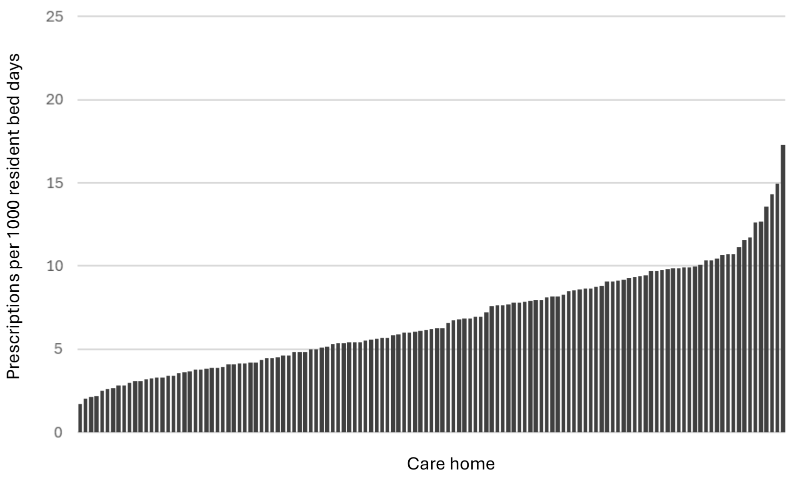
</td></tr>
<tr><td>148 care-homes with mean 6.61 (SD 3.06), range 1.04 to 20.72, prescriptions per 1000 RBD</td><td>129 care-homes with mean 6.78 (SD 3.03), range 1.72 to 17.30, prescriptions per 1000 RBD</td></tr>
</table>

References

1. National Center for Health Statistics - Centers for Disease Control and Prevention (CDC). *International Classification of Diseases,Tenth Revision (ICD-10)*. https://www.cdc.gov/nchs/icd/icd10.htm

2. *The Care Inspectorate*. https://www.careinspectorate.com/
